# Embelin, a small molecule quinone with a co-clinical power for castrate-resistant prostate cancer

**DOI:** 10.3389/fphar.2014.00184

**Published:** 2014-08-08

**Authors:** Radhika J. Poojari

**Affiliations:** Department of Biosciences and Bioengineering, Indian Institute of Technology BombayMumbai, India

**Keywords:** embelin, castrate resistant prostate cancer, integrated human-mouse cross-species, gene therapy, co-clinical

Prostate cancer is the second common leading cause of cancer deaths in men worldwide. Medical castration is the standard-care treatment for metastatic prostate cancer patients. Aggressive prostate cancers have a progressive and morbid disease process with a median survival of 9–30 months (Nabhan et al., 2011; Liu and Zhang, 2013). Androgen-deprivation therapy (ADT) puts prostate cancer in remission. Hormonal therapies help in controlling advanced prostate cancers for some time and later on fail to respond, evolve resistance mechanisms, and undergo genetic deregulations with poor patient survival rate and no cure. As truly said, “Prevention is better than cure” we look forward for tailoring new treatment paradigms for the prevention of castration-resistant prostate cancers (CRPC).

Developing new, effective treatments and understanding the genetic catastrophes behind CRPCs is very challenging. Lunardi and team from Beth Israel Deaconess Medical Center, Harvard Medical School and other institutes unveiled how a co-clinical strategy comprising of a naturally occurring hydroxybenzoquinone, Embelin which is a small molecule X-linked inhibitor of apoptosis (XIAP), in dual/triple combinations with MDV3100 an androgen receptor (AR) antagonist, Bicalutamide an antiandrogen (Casodex) or Dutasteride a SRD5A1 (encoding 3-oxo-5-α-steroid 4-dehydrogenase 1) inhibitor and ADT currently in clinical trials are the targets for CRPC (Lunardi et al., 2013). Prostate cancers are characterized by distinct genetic backgrounds which respond differentially to ADT in mice and humans (Taylor et al., 2010). Moving closer to find important answers Lunardi et al. developed an integrated human-mouse cross-species genetic screening system which lead to identification of the key molecular pathways and genetic alterations in response to the standard therapeutics and new biomarkers. They conducted an array-based comparative genomic hybridization which revealed the concomitant genetic loss and mutation of PTEN and ZBTB7A and TP53 stratifies with poor responsiveness to castration. The gene expression arrays suggested downregulation of XAF1 (X-linked inhibitor of apoptosis protein–associated factor-1), upregulation of SRD5A1, relocalization of AR to the nucleus and metastasis lead to poor sensitivity to ADT. The genetic make-up revealed the road-map of a powerful triple combination therapeutic strategy to CRPC patients genetically stratified by XAF1, XIAP, and SRD5A1. The so-called genomic triad with a co-clinical strategy is justified (Figure 1).

**Figure 1:**
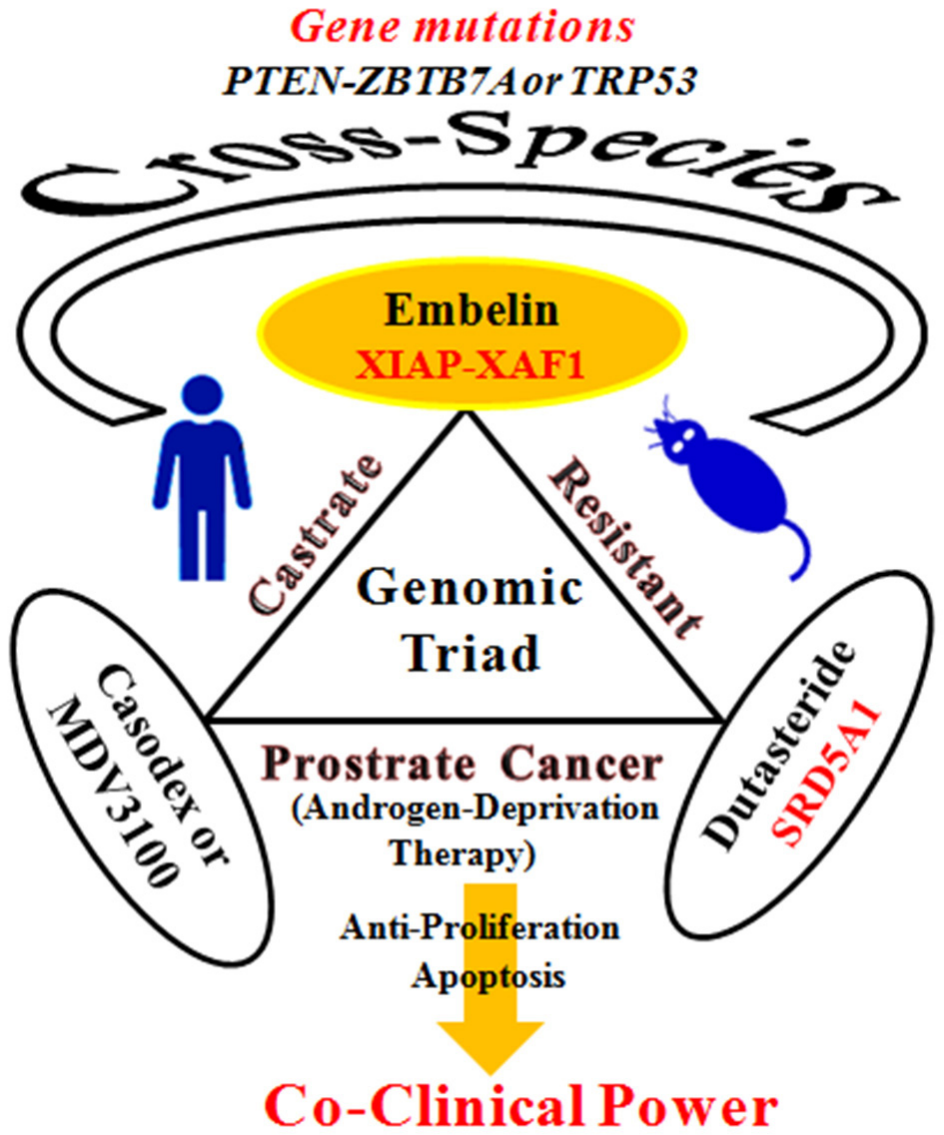
**A genomic triad with a co-clinical strategy targets for castrate-resistant prostate cancer**.

Adverse reactions such as reproductive system and breast disorders, breast tenderness, gastrointestinal disorders, hot flushes, nausea, diarrhea, hepatic disturbances (elevated transaminases levels and jaundice), erectile dysfunction, significant hypersensitivity reactions and drug resistance has been reported with antiandrogen agents like Bicalutamide, Dutasteride and MDV3100 in clinics (McLeod, 1997; Liu and Zhang, 2013). A small molecule quinone, Embelin (2,5dihydroxy-3-undecyl-1,4 benzoquinone) the major active constituent derived from the fruits (2.3%) of *Embelia* species (local name; Vidanga) has been known since antiquity in the indigenous systems of medicine well-documented for its antifertility, potent antioxidant and anti-prostate cancer properties (Danquah et al., 2012; Poojari, 2014). Embelin in combination with ionizing radiation exhibits tumor suppression and angiogenesis in hormone refractory prostate cancer resistant to radiation therapy (Dai et al., 2008). The analysis of Embelin in combination with the above indicated antiandrogens- based approach is outstanding. In the present study, human prostate cancer cells VCaP, LNCaP, C4-2, PC3, DU145, and castration-resistant mouse models with concomitant loss of PTEN-ZBTB7A and PTEN-TP53 were used. A combination of Bicalutamide (10 mg/kg) and Embelin (60 mg/kg) in 0.1% carboxymethyl cellulose oral administration daily for 5 days per week for 4 weeks were evaluated. Triple treatment combinations with Bicalutamide (10 mg/kg), Embelin (60 mg/kg) oral dose for 3 days per week, Bicalutamide (10 mg/kg) and Dutasteride (2 mg/kg) oral dose 2 days per week for four weeks were also conducted. Embelin sensitized the CRPCs to ADT via XAF1-XIAP pathway, exhibited marked tumor regression and potent reduction in the proliferation rate when treated in combination with Bicalutamide in both PTEN and ZBTB7A genotypes in CRPCs. Embelin in combination with steroid-free medium and MDV3100 triggered the apoptosis promotion. Also, double null for PTEN and ZBTB7A, for PTEN and TP53 as well as PTEN-null CRPCs with SRD5A1 upregulation and, particularly the tricombo power of Dutasteride further significantly decreased the prostate tumor burden in response to Embelin treatment and ADT (Lunardi et al., 2013).

In the era of personalized medicines for cancer-care, there is a dire need to focus on defining and treating cancer by its genetic abnormalities. In the past few decades, molecularly targeted therapies for specific tumor mutations in patients are in full swing. This meticulous research analysis is exemplary for its contribution in the field of prostate cancer genetics-based novel therapeutic modality for overcoming castration resistance. It illuminates the potency of quinonic Embelin, a rich heritage of traditional herbal medicine much unknown about its novel “co-clinical genomics mix” for the first time via the integrated cross-species genetic approach system. The abrogation of the XAF1/XIAP pathway combined with SRD5A1 inhibition and ADT, implicates this new signature in designing and promotion of a natural plant based drug-targeting therapeutics on genetic mutations-driving biomarkers for the treatment of CRPC. The power of genomic triad with a co-clinical strategy would revolutionalize into translational human cancer therapy paving the way for similar approaches to other cancers too.

## Conflict of interest statement

The author declares that the research was conducted in the absence of any commercial or financial relationships that could be construed as a potential conflict of interest.

## References

[B1] Dai Y. Liu M. Tang W. DeSano J. Burstein E. Davis M. (2008). Molecularly targeted radiosensitization of human prostate cancer by modulating inhibitor of apoptosis. Clin. Cancer. Res. 14, 7701–7710 10.1158/1078-0432.CCR-08-0188 19047096 PMC2605643

[B2] Danquah M. Duke C. B. 3rd. Patil R. Miller D. D. Mahato R. I. (2012). Combination therapy of antiandrogen and XIAP inhibitor for treating advanced prostate cancer. Pharm. Res. 29, 2079–2091 10.1007/s11095-012-0737-1 22451249

[B3] Liu J. J. Zhang J. (2013). Sequencing systemic therapies in metastatic castration-resistant prostate cancer. Cancer Control 20, 181–187 23811702 10.1177/107327481302000306

[B4] Lunardi A. Ala U. Epping M. T. Salmena L. Clohessy J. G. Webster K. A. (2013). A co-clinical approach identifies mechanisms and potential therapies for androgen deprivation resistance in prostate cancer. Nat. Genet. 45, 747–755 10.1038/ng.2650 23727860 PMC3787876

[B5] McLeod D. G. (1997). Tolerability of nonsteroidal antiandrogens in the treatment of advanced prostate cancer. Oncologist 2, 18–27 10388026

[B6] Nabhan C. Parsons B. Touloukian E. Z. Stadler W. M. (2011). Novel approaches and future directions in castration resistant prostate cancer. Ann. Oncol. 22, 1948–1957 10.1093/annonc/mdq639 21252057

[B7] Poojari R. (2014). Embelin–a drug of antiquity: shifting the paradigm towards moder medicine. Expert Opin. Investig. Drugs 23, 427–444 10.1517/13543784.2014.867016 24397264

[B8] Taylor B. S. Schultz N. Hieronymus H. Gopalan A. Xiao Y. Carver B. S. (2010). Integrative genomic profiling of human prostate cancer. Cancer Cell 18, 11–22 10.1016/j.ccr.2010.05.026 20579941 PMC3198787

